# Teachers’ Knowledge of Postural Health in Children and Adolescents: A Cross-Sectional Study Using the TBPLQ

**DOI:** 10.3390/children13060836

**Published:** 2026-06-21

**Authors:** Marta Kinga Labecka, Magdalena Plandowska, Agnieszka Jankowicz-Szymańska

**Affiliations:** 1 Faculty of Rehabilitation, Jozef Pilsudski University of Physical Education, 00-968 Warsaw, Poland; 2 Faculty of Physical Education and Health in Biala Podlaska, Jozef Pilsudski University of Physical Education in Warsaw, 21-500 Biala Podlaska, Poland; magdalena.plandowska@awf.edu.pl; 3 Department of Physiotherapy, Faculty of Medicine and Health Sciences, University of Applied Sciences in Tarnow, 33-100 Tarnow, Poland; jankowiczszymanska@gmail.com

**Keywords:** functional health, postural education, teacher training, school health promotion, musculoskeletal disorders, prevention, postural deviations

## Abstract

**Background/Objectives**: Promoting postural health in children requires not only adequate knowledge but also the implementation of health-promoting behaviors in the school environment. Teachers play a key role in this process; however, the extent to which their knowledge is reflected in everyday practice remains unclear. The study aimed to analyze and compare the levels of knowledge among preschool, early school, and physical education teachers regarding postural health in children and adolescents, including postural abnormalities, ergonomics, the selection of corrective exercises, and behaviors that promote correct body posture. **Methods**: A cross-sectional study was conducted on a sample of 153 teachers in Poland: 24 preschool (P), 53 early school education (EE), and 76 physical education (PE) teachers. The self-report Teachers’ Body Posture Literacy Questionnaire (TBPLQ) was used to assess knowledge regarding postural abnormalities. **Results**: PE achieved the highest TBPLQ scores, with significant differences observed mainly in comparison with EE (r = 0.30–0.50, *p* < 0.001). Across all groups, teachers performed best in recognizing postural abnormalities and worst in selecting appropriate corrective exercises. Although knowledge levels were relatively high, only weak correlations were found between knowledge and postural hygiene-promoting behaviors. The largest behavioral differences concerned the use of appropriate sportswear during physical education classes (η^2^ > 0.14). **Conclusions**: Teachers demonstrated relatively high levels of knowledge regarding posture health. However, a clear knowledge–behavior gap was identified. Knowledge was only partially translated into proactive health-promoting actions, particularly regarding corrective interventions and communication with parents. The results suggest the need for educational initiatives for teachers focusing on proactive health-promoting and postural hygiene behaviors.

## 1. Introduction

Postural deviations in children and adolescents are recognized as a serious, common health problem. A study by the Pediatric Orthopaedic Society of North America found a prevalence of postural deviations among 9.6 million children under 19 years of age [1]. Studies in Poland report that the incidence of postural deviations ranges from 10% to 80% [2,3]. In addition, increasing sedentary behavior and prolonged sitting in the school environment have been identified as important risk factors for low back pain in children and adolescents [4]. The discrepancies in the frequency of postural deviations are related to multiple definitions of abnormalities, the individual course of posturogenesis for each person, the adopted criteria for correct assessment of body posture, and various research methods [5].

Body posture undergoes changes throughout human development, influenced by external and internal factors [6]. Moreover, postural deviations may lead to degenerative changes, chronic pain, musculoskeletal disorders, and impaired psychosocial functioning [2,3]. Considering the above, systematic screening tests, prevention, and corrective exercises are essential at every stage of a child’s development to ensure appropriate conditions for reducing postural deviations.

One of the most important tools to prevent or minimize this prevalence is acquiring knowledge concerning postural health [6].

It is well known that knowledge alone is unlikely to be sufficient to change habits and health behaviors [7,8]. However, for habits to become a key element in improving healthcare, particularly postural health, access to knowledge should be the first step in the teaching–learning process [9]. It is widely accepted that voluntary behaviors are influenced by relevant knowledge; therefore, there is a pressing need for health promotion programs and reliable tools to assess postural habits, increase awareness and understanding of postural health, and reduce the risk of developing postural deviations in the school environment. The active involvement of teachers, parents, and students is essential to implement the necessary changes [10,11]. Teachers must act as examples and role models for students [12], especially by demonstrating good postural habits through their behavior.

According to the World Health Organization [13], schools are an ideal environment for developing health literacy (HL) because children spend much of their time there and interact regularly with peers [14]. Teachers are a fundamental element in acquiring knowledge about postural education. This is particularly important for preschool and early education teachers, who have daily and prolonged contact with children during the first sensitive phase of posturogenesis. Owing to their continuous observation of children during sitting, writing, play, and movement activities, they have an opportunity to identify early postural abnormalities and reinforce healthy postural habits. Moreover, postural education is a fundamental pillar on which appropriate physical activity and healthy habits are based; it should be developed by teachers [15]. Education can contribute to developing a healthier lifestyle [8].

Both tools assess knowledge about postural health in children [16,17,18], and educational programs promoting correct postural habits [19,20,21,22,23,24] have been well documented. Salman et al. [25] identified 21 questionnaires developed to evaluate knowledge of postural habits, ergonomics, and posture. Most of these tools were designed for university or high school students, were not properly validated, and have only moderate psychometric value. To our knowledge, this is among the first cross-sectional studies to assess such knowledge using a validated questionnaire.

Despite the growing number of studies on postural education and related interventions, there is a clear lack of research using validated, pictorial tools to assess teachers’ knowledge of postural health across different educational levels. Moreover, relatively little is known about how such knowledge translates into teachers’ self-reported behaviors in everyday school practice. Based on differences in professional training and responsibilities, it may be expected that physical education teachers will demonstrate higher levels of postural knowledge than preschool and early education teachers. At the same time, in line with previous research suggesting that knowledge alone is insufficient to change behavior, it is hypothesized that higher knowledge levels will not necessarily be strongly reflected in self-reported preventive and educational behaviors.

Therefore, this study aimed to analyze and compare the levels of knowledge among preschool, early school, and physical education teachers regarding postural health in children and adolescents, including postural abnormalities, ergonomics, the selection of corrective exercises, and behaviors that promote correct body posture.

## 2. Materials and Methods

### 2.1. Study Design

A cross-sectional study was performed.

### 2.2. Ethical Statement

The study was approved by the Ethics Committee of the AWF Warsaw (SKE 01-07/2023, date 18 February 2023) and complies with the Declaration of Helsinki. Respondents provided informed written consent. Participation in the study was voluntary and anonymous, maintaining data privacy and confidentiality.

### 2.3. Subject Population

The study population consisted of teachers from all types of schools (kindergarten, primary, secondary, and high school) in different cities in Poland. Although the study aimed to include teachers from preschool (P), early school education (EE), and physical education (PE) levels, no priori stratified sampling with predefined recruitment quotas was implemented. The sample under study was obtained using a non-randomized, convenience-based snowball sampling approach, which allowed the inclusion of teachers from different educational levels and ensured coverage of a broader educational spectrum. The study included 153 teachers: 24 preschool (P), 53 early education (EE), and 76 physical education (PE) teachers. The groups were comparable in age and work experience, with mean ages ranging from 43.6 to 47.2 years and professional experience from 16.0 to 18.2 years. The final distribution of participants across groups reflects the outcome of the recruitment process rather than predefined stratification. The sample is not probabilistically representative of the national teacher population in Poland; however, it shows some similarities in key demographic characteristics (e.g., gender and age distribution), where approximately 84% are female, and the average age is 46 years [26]; but these similarities do not indicate statistical representativeness.

### 2.4. Selection Criteria

Participants were recruited according to the following inclusion criteria: teachers of both sexes, of different ages, from various educational centers (public and private schools), and who were currently professionally active. The survey collection period was set at ten-week periods, and only the correctly completed surveys received during that time were included in the analysis. Participants were excluded from the study based on the following criteria: incorrect questionnaire completion and failure to answer all questions in the required section.

### 2.5. Instrument

To analyze knowledge regarding postural deviations, individuals were required to complete the Teachers’ Body Posture Literacy Questionnaire (TBPLQ) [27]. The Polish and English versions of the questionnaire are also available on the ePROVIDE™ platform. In the original validation study, the questionnaire demonstrated satisfactory psychometric properties, including good test–retest reliability and evidence of content validity. The tool was designed as a multidimensional, pictorial questionnaire, allowing assessment across several domains of postural literacy while maintaining accessibility across different educational and cultural contexts [26]. The tool is inclusive and cross-cultural because it contains amorphous silhouettes by age, gender, and cultural background, showing posture types and body positioning during work and leisure. This questionnaire consists of 40 questions divided into three parts: 1—postural abnormality section (four subscales used to assess the teachers’ ability to recognize, name, choose possible causes, and perform proper exercises for body abnormalities); 2—postural ergonomics section (two subscales provided to assess teachers’ ability to recognize correct posture during daily activity, and explain why it is correct); and 3—sociodemographic data section (age, gender, seniority, lifestyle, etc.). The questionnaire also includes metrics about teachers’ behaviors promoting correct body posture in children and adolescents (single-choice, multiple-choice, or Likert scale-based questions).

### 2.6. Data Collection

Data were collected for 10 weeks between January and March 2024. Respondents were recruited via posts on relevant educational forums and social media (email, Facebook, Twitter, etc.), including a link to the online questionnaire. Recruitment was a non-randomized, convenience-based snowball sampling method. As the survey was distributed via open online platforms, the total number of individuals invited to participate could not be determined. The questionnaires were completed using the Google Drive application, which was accessible on both desktop and mobile devices. Participation was anonymous, and no personal identifiers were collected. To minimize duplicate responses, each participant could submit the form only once using a unique email account, and responses were additionally screened for potential duplication during data cleaning. The researcher verified email addresses associated with submitted questionnaires and reviewed response records to identify potential duplicate entries. The backend researcher checked the online questionnaire and eliminated invalid responses.

### 2.7. Statistical Analysis

Statistical analyses were performed using Statistica version 13. Descriptive statistics for quantitative variables are presented as medians and interquartile ranges (Q1–Q3), as the data did not meet the assumption of normality according to the Shapiro–Wilk test. Between-group differences were assessed using the Kruskal–Wallis test, followed by Bonferroni-corrected post hoc comparisons when appropriate. Statistical significance was set at *p* < 0.05.

TBPLQ outcomes were analyzed using raw scores. Descriptive results are presented in points, while percentages of the maximum possible score are additionally reported in the text to facilitate interpretation and comparison across subscales with different score ranges.

Internal consistency of the TBPLQ and its subscales was evaluated using Cronbach’s alpha coefficient. The magnitude of group differences was assessed using rank eta squared (η^2^) for Kruskal–Wallis tests and Rosenthal’s correlation coefficient (*r*) for pairwise post hoc comparisons. Rank eta squared was calculated as η^2^ = (H − k + 1)/(*n* − k), where *H* is the Kruskal–Wallis statistic, *k* is the number of groups, and *n* is the total sample size. Effect sizes were interpreted as small (η^2^ > 0.01), moderate (η^2^ > 0.06), or large (η^2^ > 0.14). For Rosenthal’s *r*, values of 0.10, 0.30, and 0.50 were interpreted as small, medium, and large effects, respectively.

Associations between TBPLQ scores and posture-related behaviors were examined using Spearman’s rank correlation coefficient.

## 3. Results

Reliability analysis showed good reliability for subscale 1 (Cronbach’s α = 0.81) and the TBPLQ total score (Cronbach’s α = 0.86), as well as acceptable reliability for subscale 2 (Cronbach’s α = 0.77).

A moderate effect (η^2^ > 0.06) was observed for Section 1.3 of subscale 1, subscale 1, and the TBPLQ total score, as well as a strong effect (η^2^ > 0.14) for Section 1.2 of subscale 1.

The effect size analysis indicated moderate to large differences between early education and physical education teachers (r = 0.30–0.50), particularly in Sections 1.2 and 1.3 of Subscale 1, the total score of Subscale 1, and the overall TBPLQ score. Smaller but significant differences were observed between preschool and physical education teachers (r = 0.10–0.30) in selected sections of Subscale 1, Section 2.2 of Subscale 2, and the overall TBPLQ score (Table 1).

The most pronounced differences occurred between early education and physical education teachers regarding changing into sportswear for physical education lessons (r ≈ 0.30–0.50). Smaller differences were observed between the studied groups for active participation in exercises and ensuring appropriate sportswear (r ≈ 0.10–0.30), whereas negligible differences (r < 0.10) were found for encouraging physical activity, discussing correct posture, and including posture-related exercises during classes (Table 2).

The relationships between TBPLQ scores and responses related to behaviors promoting postural hygiene were assessed for the groups of teachers studied. Only weak correlations were observed between knowledge of postural deviations and behaviors promoting postural hygiene. The majority of significant correlations were observed in the early education teachers’ group. A negative correlation indicates that higher knowledge scores were associated with lower response values on the Likert scale. Because the behavioral items were coded from 1 (“always”) to 5 (“never”), this means that participants with greater knowledge reported engaging more frequently in behaviors promoting postural hygiene (Table 3).

## 4. Discussion

The study aimed to analyze and compare knowledge levels among preschool, early school, and physical education teachers regarding postural health in children and adolescents, including postural abnormalities, ergonomics, the selection of corrective exercises, and behaviors that promote proper posture. The research shows that, in each group of teachers, the respondents were best at interpreting graphics and worst at selecting the appropriate exercise to correct the defect. Although they demonstrated relatively high knowledge levels about postural deviations, they reported limited sharing of information about the importance of correct posture and the potential consequences of postural problems with parents and others. This reveals a clear discrepancy between teachers’ declarative knowledge and their self-reported preventive and educational behaviors in everyday practice.

To our knowledge, this is one of the first studies to measure teachers’ knowledge regarding children’s postural deviations among teachers across the three main educational stages using a validated questionnaire. Therefore, it is difficult to relate the research results to the research of other authors. Previous studies do exist; however, they often rely on non-validated tools or focus on narrower aspects of the issue. To our knowledge, there is only one study that has evaluated knowledge about postural ergonomics among teachers using a questionnaire [27]. Specifically, Jabeen and Hussain [28] investigated teachers’ awareness and practices on school ergonomics in Pakistan. The result indicated that ergonomics is highly valued in school, and teachers introduce appropriate physical exercise in the classroom to keep children healthy and active. Only five teachers at a private school were surveyed, and therefore the results should be interpreted with caution.

Previous studies have reported that teachers often possess insufficient knowledge related to posture education and the prevention of postural deviations in children, while educational programs can significantly improve their knowledge and awareness [19,20,21,22,23,24]. Furthermore, research from several countries suggests that many teachers do not feel adequately prepared to deliver health education content and may lack both content-specific and pedagogical competencies in this area [14,15]. The findings of the present study are consistent with these observations. Specifically, early education teachers (EE) demonstrated significantly lower postural knowledge than physical education teachers (PE). While EE teachers were generally able to recognize postural abnormalities and identify ergonomic positions or corrective exercises, they showed lower competence in naming postural deviations and understanding their characteristics. This gap is particularly concerning, as early education teachers play a central role during an important stage of children’s postural development, as noted in the Introduction. Therefore, there is a clear need for comprehensive initial and continuing education programs for teachers to better support student health [29]. When teachers recognize incorrect postural habits, they may contribute to identifying postural deviations and correcting the postural habits of their students [30,31].

Furthermore, teachers’ suggestions emphasize the need for a comprehensive strategy that aligns with international recommendations and actively involves all community stakeholders in the health education process. Continuous professional development focused on health education frameworks, as well as up-to-date health knowledge and pedagogical skills, is essential for maintaining teachers’ engagement in this field. Current curricula tend to favor traditional approaches to health education rather than embracing a holistic perspective [32].

In line with the recommendations of the World Health Organization and UNESCO, it is crucial to provide both pre-service and in-service training for teachers on key topics related to children’s health [33,34]. Additionally, educators should be equipped with participatory and proactive teaching methodologies that effectively communicate health-related content. This approach fosters personal internalization of health knowledge among students and encourages them to take practical actions toward healthy lifestyles, such as balanced nutrition and regular physical activity. Health education should be integrated into school curricula either within scientific disciplines or as a standalone subject offered through extracurricular activities, under the direct guidance of school staff [35].

### 4.1. Implications for Practice

1. Develop and implement comprehensive training programs that combine theoretical knowledge with practical application for teachers, especially for preschool and early school. These programs should include the basics of posture screening, recognition of common postural abnormalities, selection of appropriate corrective exercises, and effective communication with parents regarding observed postural concerns. They should also expand knowledge of posture and corrective practices, while building intrinsic motivation and a sense of responsibility to actively promote postural hygiene among children.
2. Ensure that training includes practical strategies for integrating posture-promoting habits into daily educational routines, including classroom ergonomics (e.g., appropriate desk and chair use, sitting habits, and backpack management), movement breaks, and age-appropriate physical activities, rather than focusing solely on theoretical knowledge.
3. Support teachers in preventing musculoskeletal disorders in themselves and their students by strengthening competencies related to posture assessment, corrective exercise planning, ergonomic classroom organization, and collaboration with parents and healthcare professionals when postural abnormalities are identified. Strengthening their competence in this area will contribute to long-term health outcomes and help foster healthier future generations.

### 4.2. Strengths

One of the key strengths of this study is its inclusion of teachers from various types of schools (kindergarten, primary, secondary, and high school) and educational levels (preschool, early school, and physical education), which enhances the diversity of the sample and supports the broader relevance of the findings rather than strong generalizability. Additionally, the demographic characteristics of the sample closely match national statistics, particularly in terms of age and gender distribution, which may support external validity to some extent, although this should be interpreted with caution given the non-random sampling approach. Another important strength is the use of the validated and reliable Teachers’ Body Posture Literacy Questionnaire (TBPLQ). The instrument not only assesses knowledge related to postural abnormalities and ergonomics but also evaluates teachers’ behaviors in promoting correct posture among students, providing a multifaceted view of postural literacy.

### 4.3. Limitations

Despite these strengths, the study has several limitations. The actual participant recruitment was conducted through non-randomized, convenience-based snowball sampling via online platforms. This method might have limited the sample’s representativeness by introducing self-selection bias. Teachers with a greater interest in postural health or higher confidence in their knowledge may have been more likely to participate, whereas others may have chosen not to respond. Consequently, the findings may overestimate the level of knowledge and engagement with postural health among the broader teacher population. In addition, because the questionnaire was administered exclusively online, teachers with limited internet access or lower digital literacy may have been underrepresented, potentially introducing digital bias. Although measures were taken to verify respondent eligibility and identify duplicate responses, selection bias could not be fully eliminated.

Moreover, as the study relied on self-reported data, the results may be influenced by social desirability bias. The behavioral items in the questionnaire were primarily descriptive and heterogeneous in format, which limited the possibility of constructing a unified behavioral score for overall behavioral assessment. While associations between knowledge and individual behavioral items were examined using Spearman correlation analysis, the heterogeneity of these items limits the interpretation and comparability of the observed relationships. Therefore, these correlations should be interpreted cautiously and should not be regarded as a comprehensive measure of teachers’ postural health behaviors. In addition, the analyses were primarily based on group comparisons and bivariate associations. Multivariable regression modeling was not performed; therefore, potential confounding effects of variables such as age, professional seniority, or prior training could not be fully disentangled from differences observed between teacher groups. The cross-sectional design also precludes any inference about causality or long-term changes in teachers’ postural knowledge or practices.

Future studies should use randomized or stratified cluster sampling based on selected schools to improve representativeness. Future research should also employ multivariable analytical approaches to evaluate the independent contribution of teacher characteristics and professional experience to postural knowledge and behaviors. Furthermore, given the number of statistical comparisons performed, the findings should be interpreted with caution and treated as partly exploratory, despite the application of the Bonferroni adjustment.

## 5. Conclusions

Preschool, early school, and physical education teachers demonstrated relatively high levels of knowledge regarding postural health. However, this knowledge was only partially reflected in preventive and health-promoting practices. For example, only approximately half of the respondents reported informing parents about identified postural abnormalities, and engagement in independent posture assessment remained limited.

These findings highlight a gap between knowledge and practice and underscore the need for both theoretical and practical teacher training. Educational initiatives should focus not only on improving knowledge of postural health but also on strengthening teachers’ motivation, confidence, and ability to implement posture-promoting strategies in everyday school settings. Particular emphasis should be placed on developing practical competencies that can be integrated into classroom routines and children’s daily activities.

Investing in teacher education may help prevent musculoskeletal disorders, support teachers’ well-being, and promote healthier outcomes for future generations, especially when training programs combine knowledge acquisition with practical skill development.

## Figures and Tables

**Table 1:** Teachers’ knowledge of postural deviations.

Variable	Preschool Teachers (P) *n* = 24	Early Education Teachers (EE) *n* = 53	Physical Education Teachers (PE) *n* = 76	Kruskal–Wallis Test and Post Hoc Bonferroni Correction (If Needed)	η^2^	R
Subscale 1.1—recognize faulty posture (max. 8 pts) Me (Q1–Q3)	8.00 (7.00–8.00)	8.00 (7.00–8.00)	8.00 (8.00–8.00)	H (df = 2) = 4.82; *p* = 0.090	0.02	P vs. EE r = 0.01 P vs. PE r = 0.13 EE vs. PE r = 0.16
Subscale 1.2—name the postural abnormality identified (max. 8 pts) Me (Q1–Q3)	7.00 (4.00–8.00)	5.00 (4.00–7.00)	7.00 (6.00–8.00)	H (df = 2) = 27.618; *p* < 0.001 * P vs. EE *p* = 0.133 P vs. PE *p* = 0.432 EE vs. PE *p* = 0.001 *	0.17	P vs. EE r = 0.11 P vs. PE r = 0.24 EE vs. PE r = 0.42
Subscale 1.3—select the statement that is correct in your opinion regarding this postural abnormality (max. 8 pts) Me (Q1–Q3)	5.00 (3.25–6.00)	5.00 (4.00–6.00)	6.00 (5.00–7.00)	H (df = 2) = 16.107; *p* < 0.001 * P vs. EE *p* = 1.000 P vs. PE *p* = 0.054 EE vs. PE *p* = 0.002 *	0.09	P vs. EE r = 0.03 P vs. PE r = 0.19 EE vs. PE r = 0.29
Subscale 1.4—choose the best exercise to correct your posture defect (max. 8 pts) Me (Q1–Q3)	4.00 (3.00–5.00)	4.00 (3.00–5.00)	4.50 (3.00–5.00)	H (df = 2) = 4.181; *p* = 0.124	0.01	P vs. EE r = 0.09 P vs. PE r = 0.05 EE vs. PE r = 0.19
Subscale 1. TOTAL max 32 pts Me (Q1–Q3)	23.00 (17.00–26.75)	22.00 (18.50–24.00)	25.50 (22.25–28.00)	H (d f= 2) = 21.182; *p* < 0.001 * P vs. EE *p* = 0.972 P vs. PE *p* = 0.050 EE vs. PE *p* < 0.001 *	0.13	P vs. EE r = 0.09 P vs. PE r = 0.21 EE vs. PE r = 0.37
Subscale 2.1—select the correct body position adopted during daily activities (max. 8 points) Me (Q1–Q3)	8.00 (7.00–8.00)	8.00 (7.00–8.00)	8.00 (7.25–8.00)	H (df = 2) = 4.761; *p* = 0.092	0.02	P vs. EE r = 0.11 P vs. PE r = 0.05 EE vs. PE r = 0.20
Subscale 2.2—explain why you think this body position is correct (max. 8 pts) Me (Q1–Q3)	7.00 (6.00–7.00)	6.00 (5.00–8.00)	7.00 (6.00–8.00)	H (df = 2) = 6.030; *p* = 0.049 * P vs. EE *p* = 1.000 P vs. PE *p* = 0.604 EE vs. PE *p* = 0.050	0.03	EP vs. EE r = 0.08 P vs. PE r = 0.10 EE vs. PE r = 0.24
Subscale 2. TOTAL max 16 pts Me (Q1–Q3)	15.00 (14.00–15.00)	14.00 (12.00–15.00)	15.00 (14.00–16.00)	H (df = 2) = 8.183; *p* = 0.017 * P vs. EE *p* = 1.000 P vs. PE *p* = 0.604 EE vs. PE *p* = 0.050	0.04	P vs. EE r = 0.10 P vs. PE r = 0.09 EE vs. PE r = 0.26
TBPLQ TOTAL max 48 pts Me (Q1–Q3)	38.00 (31.00–42.00)	36.00 (30.50–39.50)	40.00 (37.25–42.75)	H (df = 2) = 21.191; *p* < 0.001 * P vs. EE *p* = 0.565 P vs. PE *p* = 0.109 EE vs. PE *p* < 0.001 *	0.13	P vs. EE r = 0.01 P vs. PE r = 0.19 EE vs. PE r = 0.37

η^2^—Rank eta square; r—Rosenthal’s correlation coefficient for Bonferroni-corrected pairwise contrasts. * Statistical significance was set at *p* < 0.05.

**Table 2:** Teachers’ responses related to behaviors promoting postural hygiene (1—always, 2—often, 3—sometimes, 4—rarely, 5—never).

Variable	Group	Median	Q1	Q3	Kruskal–Wallis Test and Post Hoc Bonferroni Correction (If Needed)	η^2^	R
Do you encourage your students to engage in extracurricular physical activity for at least 60 min a day?	P	2.00	1.00	2.00	H (df = 2) = 0.038; *p* = 0.981	0.01	P vs. EE r = 0.01 P vs. PE r = 0.01 EE vs. PE r = 0.01
	EE	2.00	1.00	2.00			
	PE	2.00	1.00	2.00			
Do you pay attention to your students’ posture when they are sitting, standing, and moving?	P	1.00	1.00	2.00	H (df = 2) = 6.168; *p* = 0.046 * P vs. EE *p* = 0.582 P vs. PE *p* = 0.010 * EE vs. PE *p* < 0.001 *	0.08	P vs. EE r = 0.10 P vs. PE r = 0.18 EE vs. PE r = 0.09
	EE	1.00	1.00	2.00			
	PE	2.00	1.00	2.00			
Do you talk to your students about correct posture and the benefits of maintaining it?	P	1.00	1.00	2.00	H (df = 2) = 1.780; *p* = 0.411	0.01	EP vs. EE r = 0.08 P vs. PE r = 0.10 EE vs. PE r = 0.02
	EE	2.00	1.00	2.00			
	PE	2.00	1.00	2.00			
Do you independently assess your students’ posture?	P	3.00	2.00	4.00	H (df = 2) = 1.902; *p* = 0.386	0.01	P vs. EE r = 0.03 P vs. PE r = 0.12 EE vs. PE r = 0.12
	EE	3.00	2.00	4.00			
	PE	3.00	2.00	3.00			
Do you include exercises in your classes to reinforce the habit of correct posture?	P	2.00	1.00	2.00	H (df = 2) = 0.401; *p* = 0.818	0.01	P vs. EE r = 0.05 P vs. PE r = 0.03 EE vs. PE r = 0.04
	EE	2.00	1.00	3.00			
	PE	2.00	1.00	2.00			
Does your school support or encourage teachers to improve their knowledge about posture and their ability to assess it?	P	3.00	2.00	3.00	H (df = 2) = 4.393; *p* = 0.111	0.02	P vs. EE r = 0.03 P vs. PE r = 0.09 EE vs. PE r = 0.16
	EE	3.00	2.00	4.00			
	PE	3.00	2.00	4.00			
Do you change into sportswear for physical education lessons with students?	P	2.00	1.00	4.00	H (df = 2) = 70.074; *p* < 0.001 * P vs. EE *p* = 1.000 P vs. PE *p* < 0.001 * EE vs. PE *p* < 0.001 *	0.45	P vs. EE r = 0.13 P vs. PE r = 0.41 EE vs. PE r = 0.61
	EE	3.00	2.00	4.00			
	PE	1.00	1.00	1.00			
Do you actively do exercises with students during lessons?	P	1.00	1.00	2.00	H (df = 2) = 8.646; *p* = 0.013 * P vs. EE *p* = 0.193 P vs. PE *p* = 1.000 EE vs. PE *p* = 0.503	0.04	P vs. EE r = 0.25 P vs. PE r = 0.18 EE vs. PE r = 0.12
	EE	2.00	1.50	3.00			
	PE	2.00	2.00	3.00			
Do you ensure that students’ sportswear is appropriate?	P	2.00	1.00	2.00	H (df = 2) = 18.213; *p* < 0.001 * P vs. EE *p* = 0.108 P vs. PE *p* < 0.001 * EE vs. PE *p* = 0.042 *	0.11	P vs. EE r = 0.01 P vs. PE r = 0.26 EE vs. PE r = 0.17
	EE	1.00	1.00	2.00			
	PE	1.00	1.00	1.00			

η^2^—Rank eta square; r—Rosenthal’s correlation coefficient for Bonferroni-corrected pairwise contrasts. * Statistical significance was set at *p* < 0.05. Behavioral items were scored on a 5-point Likert scale, where 1 = “always” and 5 = “never”. Therefore, lower scores indicate more frequent engagement in posture-promoting behaviors.

**Table 3:** Correlations between TBPLQ scores and teachers’ self-reported posture-related behaviors.

Correlated Variables	Subscale 1. TOTAL			Subscale 2. TOTAL			TBPLQ TOTAL		
	P *n* = 24	EE *n* = 53	PE *n* = 76	P *n* = 24	EE *n* = 53	PE *n* = 76	P *n* = 24	EE *n* = 53	PE *n* = 76
Do you encourage your students to engage in extracurricular physical activity for at least 60 min a day?	ns	ns	ns	ns	ns	R = 0.24; *p* = 0.040	ns	ns	ns
Do you pay attention to your students’ posture when they are sitting, standing, and moving?	ns	ns	ns	ns	ns	ns	ns	R = −0.27; *p* = 0.045	ns
Do you talk to your students about correct posture and the benefits of maintaining it?	ns	R = −0.31; *p* = 0.024	ns	ns	R = −0.48; *p* < 0.001	ns	ns	R = −0.39; *p* = 0.003	ns
Do you independently assess your students’ posture?	ns	ns	ns	ns	ns	ns	ns	ns	ns
Do you include exercises in your classes to reinforce the habit of correct posture?	ns	ns	ns	ns	ns	ns	ns	ns	ns
Does your school support or encourage teachers to improve their knowledge about posture and their ability to assess it?	ns	ns	ns	ns	ns	ns	ns	ns	ns
Do you change into sportswear for physical education lessons with students?	ns	ns	ns	ns	ns	ns	ns	ns	ns
Do you actively do exercises with students during lessons?	ns	ns	ns	ns	ns	ns	ns	ns	ns
Do you ensure that students’ sportswear is appropriate?	ns	R = −0.32; *p* = 0.018	ns	ns	ns	ns	ns	R = −0.33; *p* = 0.015	ns

P—preschool teachers, EE—early education teachers, PE—physical education teachers, ns—not statistically significant, R—Spearman correlation. Statistical significance was set at *p* < 0.05.

## Data Availability

The original contributions presented in this study are included in the article. Further inquiries can be directed to the corresponding author.

## References

[1] Rusnák R., Kolarová M., Aštaryová I., Kutiš P. (2019). Screening and early identification of spinal deformities and posture in 311 children: Results from 16 districts in Slovakia. Rehabil. Res. Pract..

[2] Baranowska A., Sierakowska M., Owczarczuk A., Olejnik B.J., Lankau A., Baranowski P. (2023). An analysis of the risk factors for postural defects among early school-aged children. J. Clin. Med..

[3] Dop D., Pădureanu V., Pădureanu R., Niculescu S.-A., Drăgoescu A.N., Moroșanu A., Mateescu D., Niculescu C.E., Marcu I.R. (2024). Risk factors involved in postural disorders in children and adolescents. Life.

[4] Calvo-Muñoz I., García-Moreno J.M., Gómez-Conesa A., López-López J.A. (2026). Sedentary Behavior and Low Back Pain in Children and Adolescents: A Systematic Review and Meta-Analysis. Healthcare.

[5] Woynarowska B., Oblacińska A. (2014). Stan zdrowia dzieci i młodzieży w Polsce: Najważniejsze problemy zdrowotne. Stud. BAS.

[6] Miñana-Signes V., Monfort-Pañego M. (2022). Back-health-related physical activity and exercise knowledge in adolescents: A cross-sectional study. Children.

[7] Dugan J.E. (2018). Teaching the body: A systematic review of posture interventions in primary schools. Educ. Rev..

[8] Ennis C.D. (2007). 2006 CH McCloy Research Lecture: Defining learning as conceptual change in physical education and physical activity settings. Res. Q. Exerc. Sport.

[9] Hotham S., Hamilton-West K.E., Hutton E., King A., Abbott N. (2017). A study into the effectiveness of a postural care training programme aimed at improving knowledge, understanding and confidence in parents and school staff. Child Care Health Dev..

[10] Limon S., Valinsky L.J., Ben-Shalom Y. (2004). Children at risk: Risk factors for low back pain in the elementary school environment. Spine.

[11] Lipkin P.H., Macias M.M. (2020). Council on Children with Disabilities, Section on Developmental and Behavioral Pediatrics. Promoting optimal development: Identifying infants and young children with developmental disorders through developmental surveillance and screening. Pediatrics.

[12] Harbour K.E., Evanovich L.L., Sweigart C.A., Hughes L.E. (2015). A brief review of effective teaching practices that maximize student engagement. Prev. Sch. Fail..

[13] World Health Organization (1986). The Ottawa Charter for Health Promotion.

[14] Sørensen K., Van den Broucke S., Fullam J., Doyle G., Pelikan J., Slonska Z., Brand H. (2012). (HLS-EU) Consortium Health Literacy Project European. Health literacy and public health: A systematic review and integration of definitions and models. BMC Public Health.

[15] Chacón-Borrego F., Ubago-Jiménez J.L., La Guardia García J.J., Padial Ruz R., Cepero González M. (2018). Educación e higiene postural en Educación Física. Papel del maestro en la prevención de lesiones: Revisión sistemática. Retos.

[16] Noll M., Candotti C.T., Vieira A., Loss J.F. (2013). Back pain and body posture evaluation instrument (BackPEI): Development, content validation and reproducibility. Int. J. Public Health.

[17] Monfort-Pañego M., Miñana-Signes V. (2020). Psychometric study and content validity of a questionnaire to assess back-health-related postural habits in daily activities. Meas. Phys. Educ. Exerc. Sci..

[18] Feng D., Zhang Y. (2023). College students’ knowledge, attitudes, and practices regarding body posture: A cross-sectional survey—Taking a university in Wuhu City as an example. Prev. Med. Rep..

[19] Helmy A.A., Mohammed A.A., Yossif H.A.E. (2019). Knowledge and practices of school children and teachers regarding musculoskeletal problems: An educational intervention. Egypt. J. Health Care.

[20] Cardon G., De Bourdeaudhuij I., De Clercq D. (2002). Knowledge and perceptions about back education among elementary school students, teachers, and parents in Belgium. J. Sch. Health.

[21] Gómez A.C. Postural hygiene questionnaire to back care: Preliminary study with children. Proceedings of the 14th International Congress of the WCPT.

[22] Minghelli B. (2022). Postural habits in adolescents: Influence of school physiotherapy program on improving the knowledge of postures. Int. J. Adolesc. Med. Health.

[23] Vidal-Conti J., Galmes-Panades A.M. (2025). Effects of an online school-based intervention on postural education knowledge among teachers. Health Educ. J..

[24] Wade M.T. (2018). Effectiveness of a Posture Education Program to Increase Teacher Knowledge. Doctoral Dissertation.

[25] Salman M., Bettany-Saltikov J., Kandasamy G., Aristegui Racero G. (2024). Development of a novel pictorial questionnaire to assess knowledge and behaviour on ergonomics and posture as well as musculoskeletal pain in university students: Validity and reliability. Healthcare.

[26] Ministry of National Education (2024). MEN: 84% Polskich Nauczycieli to Kobiety. Samorząd PAP.

[27] Labecka M.K., Jankowicz-Szymańska A., Plandowska M., Olszewska E., Rajabi R. (2024). The test–retest reliability of a body posture literacy questionnaire among Polish teachers from different educational levels. PeerJ.

[28] Jabeen R., Hussain N. (2022). Teachers’ awareness and practices on school ergonomics in Karachi, Pakistan. J. Humanit. Soc. Manag. Sci..

[29] Menotti J., Justin E., Bandeira A., Menotti L.V., de Freitas Thomazi C.P., Corrêa P.S., Galvan T.C. (2018). A importância da educação postural em crianças e adolescentes. Rev. Perspect. Cienc. Saude.

[30] Dullien S., Grifka J., Jansen P. (2018). Cluster-randomized, controlled evaluation of a Teacher-led multi factorial school-based back education program for 10- to 12-year-olds. BMC Pediatr..

[31] Junior F.U.A., da Roza Paz C., Lopes R.L. (2021). Dor e desvios posturais em crianças e adolescentes: Revisão. Higia.

[32] El Kazdouh H., El-Ammari A., Bouftini S., El Fakir S., El Achhab Y. (2022). Teachers’ perceptions of health education and middle school curriculum: A qualitative study. Teach. Teach. Educ..

[33] World Health Organization (2021). Health Promoting Schools: An Effective Approach for Early Action on Noncommunicable Diseases.

[34] UNESCO (2018). Skills-Based Health Education Including Life Skills: An Important Component of a Child-Friendly, Health-Promoting School.

[35] Pulimeno M., Piscitelli P., Miani A., Colao A., Colazzo S. (2020). Training teachers as health promoters. J. Interdiscip. Res. Appl. Med..

